# Host Protein Palmitoylation: A Two-Edged Sword in the Pathogenesis of COVID-19

**DOI:** 10.3390/microorganisms14102353

**Published:** 2026-10-14

**Authors:** Shiyi Wang, Wei Yu

**Affiliations:** 1 Institute of Biochemistry, College of Life Sciences and Medicine, Zhejiang Sci-Tech University, Hangzhou 310018, China; 2025210901080@mails.zstu.edu.cn; 2 Zhejiang Provincial Key Laboratory of Silkworm Bioreactor and Biomedicine, Hangzhou 310018, China

**Keywords:** SARS-CoV-2, COVID-19, palmitoylation, ZDHHC, targeted therapy

## Abstract

Protein palmitoylation is a two-edged sword in regulating Severe Acute Respiratory Syndrome Coronavirus 2 (SARS-CoV-2) infection. On the one hand, SARS-CoV-2 hijacks the host’s palmitoyl transferases (ZDHHC5/9/20) to modify its spike protein (S protein), enhancing its stability, membrane anchoring, and fusion, consequently facilitating SARS-CoV-2 entry, replication, and release. On the other hand, the host utilises this modification to activate antiviral defences: palmitoylation of the mitochondrial antiviral signalling protein (MAVS) leads to its accumulation in the mitochondria and initiates interferon signalling; palmitoylation of the stimulator of interferon genes (STING) is essential for its activation and transport; and palmitoylation of interferon-induced transmembrane protein 3 (IFITM3) increases its ability to inhibit SARS-CoV-2 membrane fusion. This defensive–offensive interplay within the same regulatory system offers fresh opportunities for targeted therapy, including the development of specific ZDHHC inhibitors, palmitoylated angiotensin-converting enzyme 2 (ACE2)-based decoy vesicles, and metabolic intervention approaches. In the future, it will be necessary to conduct in-depth analyses of the functions of key modification sites and develop precise regulatory strategies to address the ongoing threat posed by coronaviruses.

## 1. Introduction

The COVID-19 pandemic of the past few years, with its far-reaching effects on global health, the economy, and society, has fully exposed the vulnerability of human society to infectious diseases and stressed the urgent need to empower public health systems with innovative tools [1,2,3]. Against this backdrop, it is important to gain an in-depth understanding of the molecular mechanisms underlying the interaction between pathogens and hosts [4]. Post-translational modifications (PTMs) of proteins are a key mechanism that exerts substantial effects on protein function, stability, localisation, and interactions by reversibly attaching chemical groups or complex molecules to amino acid side chains, or by irreversibly altering the amino acid sequence, thereby enabling cells to respond rapidly and reversibly to external changes [5].

In host–pathogen interactions, PTM signals have served as a focal point of competition between the two parties [6]. Host cells use a network of PTMs to regulate their behaviour and precisely fight infection; at the same time, pathogens have evolved strategies to hijack the host’s PTM system to survive and replicate within the cell [7]. In particular, intracellular bacteria can act directly as “writers” or “erasers” by secreting effector proteins into the host cell, thereby interfering with the modification of host proteins and reshaping cellular signalling pathways and physiological states [8,9,10]. These mechanisms are closely linked to the infection process and disease progression; therefore, investigations into pathways that regulate PTMs not only help explain the mechanisms underlying infectious diseases but also furnish a key conceptual basis and potential targets for the development of novel intervention approaches [5]. Metabolic homeostasis is a key factor in maintaining life balance, and metabolic reprogramming is a driven, adaptive response of cells to external or internal stimuli [11,12]. In this process, certain intermediate metabolites have a vital role in cellular signal transduction, acting as cofactors or donors for post-translational protein modifications, thereby modulating various pathophysiological processes [13]. As one of the most abundant and highly reversible protein terminal modifications, protein palmitoylation is not only extensively involved in regulating protein function. It is closely linked to lipid metabolism and cellular energy balance, thereby serving a crucial role in metabolic and signalling regulatory networks [14].

**Figure 1:**
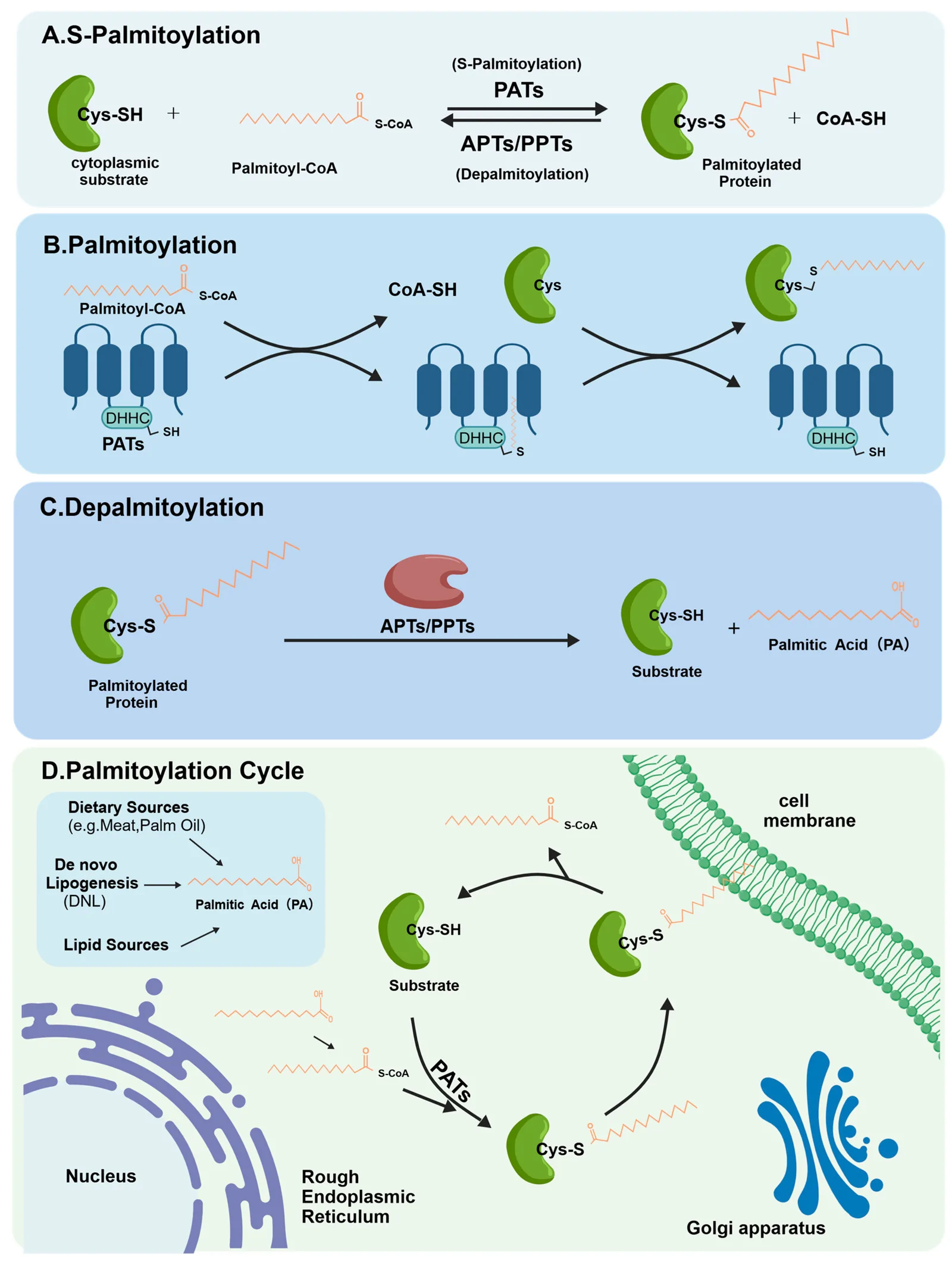
Molecular mechanisms and metabolic context of protein palmitoylation. (**A**) Reversible S-palmitoylation cycle. Under the catalysis of palmitoyltransferases (PATs), palmitoyl-CoA attaches a palmitoyl group to a cysteine (Cys) on a substrate via a thioester bond. This modification is reversed by acyl-protein thioesterases (APTs/PPTs). (**B**) Catalytic model of a DHHC-type palmitoyltransferase. The DHHC (Asp-His-His-Cys) domain’s conserved cysteine first “accepts” the palmitoyl group from palmitoyl-CoA, forming a palmitoyl–enzyme intermediate. It then “transfers” the group to the target protein’s cysteine. (**C**) Mechanism of action of depalmylases. APTs (such as PPT1) and PPTs (such as APT1/2) use their active-site cysteine to nucleophilically attack the thioester bond on the palmitoylated protein, hydrolysing it and releasing free palmitic acid (PA). (**D**) The palmitoylation cycle in context. Palmitic acid (PA) is derived from diet or de novo lipogenesis (DNL). Activated palmitoyl-CoA is used by PATs in the ER and Golgi to modify proteins. This adds hydrophobicity, driving targeted protein trafficking to the plasma membrane and other cellular membrane structures. APTs/PPTs dynamically remove this signal. Note: PATs, palmitoyl acyltransferases; APTs, acyl protein thioesterases; PPTs, palmitoyl protein thioesterases; DHHC, Asp-His-His-Cys conserved motif; PA, palmitic acid; DNL, de novo lipogenesis; ER, endoplasmic reticulum.

S-palmitoylation is a reversible post-translational lipid modification of proteins in which a 16-carbon saturated fatty acid (palmitic acid) is covalently attached to a cysteine residue on the protein via an unstable thioester bond [15] (Figure 1A). This active cycle is catalysed by palmitoyl transferases (PATs), also known as a specific family of zinc finger DHHC domain-containing protein palmitoyl transferases (ZDHHCs) (Figure 1B). In contrast, depalmylation is catalysed by acyl-protein thioesterases (APTs) and palmitoyl protein thioesterases (PPTs) [16] (Figure 1C). Palmitic acid (PA), a common saturated fatty acid, is widely found in the human body; it is not only present in foods such as palm oil and meat but is also produced through the de novo lipogenesis (DNL) process [17]. Metabolically, palmitic acid can participate in β-oxidation to provide energy, or it can be converted into phospholipids and sphingolipids, which constitute essential parts of cell membranes [18]. This modification process is catalysed by ZDHHCs [19]. Screening experiments indicate that several ZDHHC members promote the palmitoylation of spike (S) proteins, with zDHHC5, zDHHC9, and zDHHC20 likely playing a major role [20]. For example, zDHHC9 interacts with S proteins in the endoplasmic reticulum and Golgi apparatus and catalyses their palmitoylation; knocking down the zDHHC9 gene reduces the fusion and infection capabilities of Severe Acute Respiratory Syndrome Coronavirus 2 (SARS-CoV-2) [21] (Figure 1D). The use of palmitoylation inhibitors (such as 2-BP) effectively inhibits the palmitoylation of S protein [22]. By increasing the hydrophobicity of proteins, palmitoylation considerably influences their stability, conformation, subcellular localisation, membrane association, and interactions with other molecules, thereby modulating various cellular physiological processes and serving as a key switch for controlling protein function [16].

SARS-CoV-2 is the pathogen responsible for causing coronavirus disease 2019 (COVID-19). It belongs to the genus Beta coronaviruses and, along with SARS-CoV and MERS-CoV, is one of the zoonotic viruses that have caused severe pneumonia in this century; all three originated in bats. Studying their biological characteristics and mechanisms of infection is important for addressing current and future pandemics [23]. SARS-CoV-2 is an enveloped, single-stranded positive-sense RNA virus, and the S protein on its surface plays a key role in infection [24]. The S protein binds to the ACE2 receptor on the surface of host cells with high affinity via its receptor-binding domain [25]. Subsequently, under the action of transmembrane serine proteases (such as TMPRSS2), the S protein is cleaved and activated, mediating fusion of the viral envelope with the cell membrane, allowing the viral genetic material to enter the cytoplasm, and initiating infection [23].

Palmitoylation is a common modification mechanism for SARS-CoV-2 envelope proteins; it promotes membrane binding and anchoring by regulating protein interactions and enhancing protein hydrophobicity [26]. SARS-CoV-2 can also exploit this mechanism to induce palmitoylation of its S protein, thereby regulating fusion and infection at the host cell membrane [27]. Host palmitoyl transferases are involved in SARS-CoV-2 assembly, and related enzyme inhibitors can reduce SARS-CoV-2 replication, suggesting that targeting palmitoylation is a potential antiviral strategy, as palmitoylation is important in the SARS-CoV-2 life cycle and the host immune response [28]. Therefore, this paper aims to systematically explain the bidirectional regulatory role of palmitoylation during SARS-CoV-2 infection and to investigate potential clinical strategies based on this evidence, consequently providing a conceptual basis for understanding SARS-CoV-2–host interactions as well as developing novel antiviral approaches.

## 2. SARS-CoV-2 Hijacks Palmitoylation for Its Own Benefit

### 2.1. Entry

The S protein on the surface of the COVID-19 virus specifically binds to the ACE2 receptor on the host cell membrane [29]. The S protein consists of two subunits, S1 and S2; the receptor-binding domain (RBD) on the S1 subunit is responsible for recognising and binding to ACE2 [30]. Subsequently, the S protein is cleaved by host proteases at a specific cleavage site at the S1-S2 junction and may undergo glycosylation, thereby activating the S2 subunit. The activated S2 subunit mediates fusion between the viral envelope and the host cell membrane via its HR1 and HR2 domains (heptad repeats 1 and 2), resulting in the release of the viral genomic RNA into the cytoplasm [28].

The palmitoylation sites of the SARS-CoV-2 S protein are distributed at both the N-terminus and the C-terminal cytoplasmic tail. Specifically, C15 at the N-terminus serves as one palmitoylation site [31], while the C-terminal cytoplasmic tail is rich in cysteine residues and contains multiple potential palmitoylation sites, specifically C1235, C1236, C1240, C1241, C1243, C1247, C1248, C1250, C1253, and C1254 [21,32]. Experiments have shown that mutating all cysteine residues at the 10 sites mentioned above markedly lowers palmitoylation levels. Among these sites, cysteines located near the cell membrane (such as C1235 and C1236) play a dominant role in palmitoylation, and their modification may facilitate the palmitoylation of cysteines further away [32]. Specifically, C1235, C1236, C1240, C1241, C1247, and C1248 were identified as sites of high-level palmitoylation [21].

**Figure 2:**
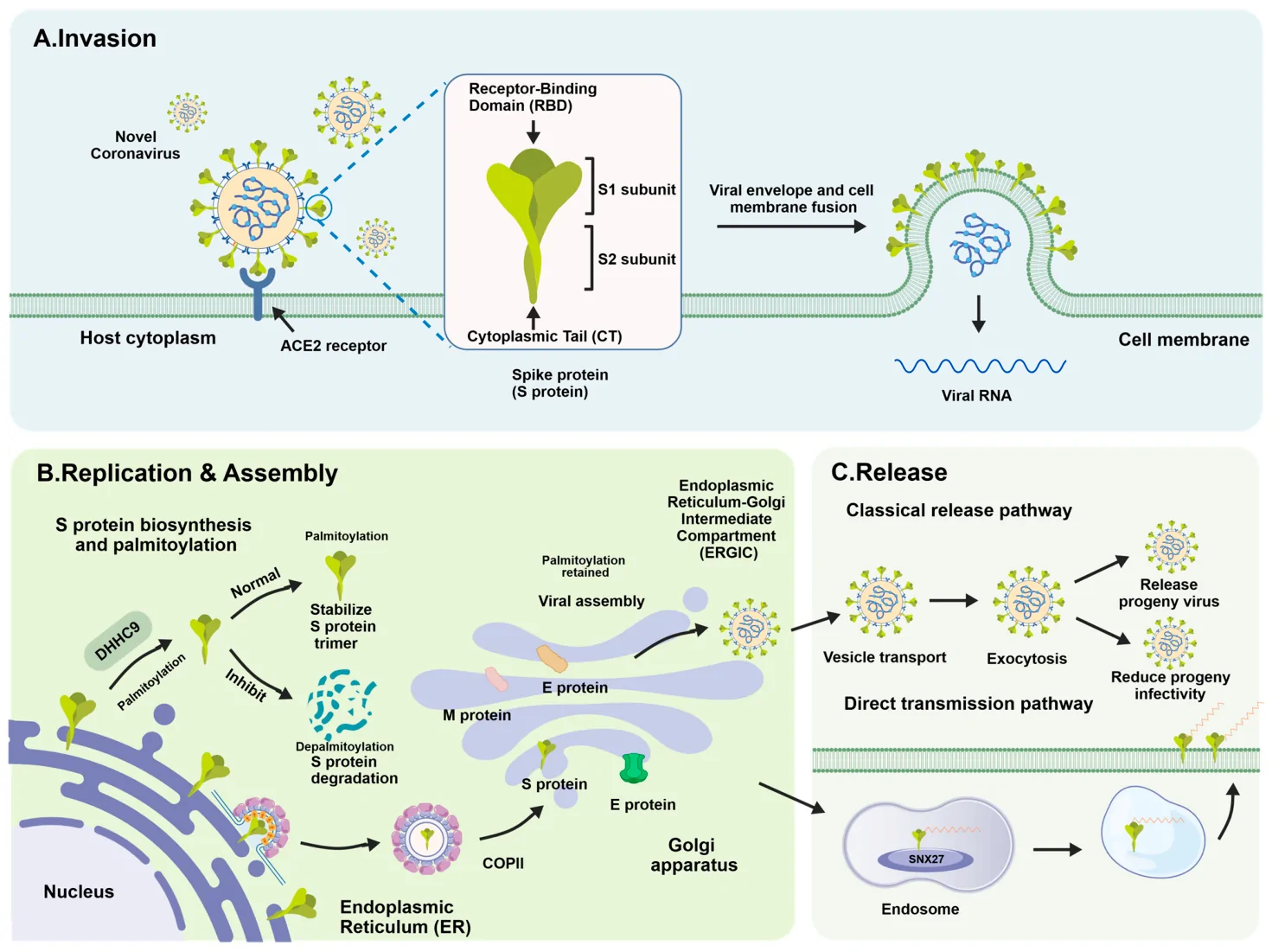
Life cycle of SARS-CoV-2. (**A**) Invasion. SARS-CoV-2 binds to the host ACE2 receptor via the receptor-binding domain (RBD) of its spike (S) protein. Under the action of the S1 and S2 subunits, the viral envelope fuses with the cell membrane, subsequently releasing viral RNA into the host cytoplasm. (**B**) Replication and assembly. The S protein is synthesised and palmitoylated in the endoplasmic reticulum (ER). Viral assembly occurs at the ER–Golgi intermediate compartment (ERGIC), where the E, M, and S proteins work cooperatively. The virus is then transported via COPII vesicles to the Golgi apparatus for further processing. (**C**) Release. Viruses are released through two pathways: (i) the classical release pathway, in which viruses are released via vesicular transport and exocytosis while reducing progeny infectivity; and (ii) the direct transmission pathway, in which viruses are released through the endosomal route, a process regulated by SNX27. Note: RBD, receptor-binding domain; ER, endoplasmic reticulum; ERGIC, ER–Golgi intermediate compartment; COPII, coat protein complex II; SNX27, sorting nexin 27.

Palmitoylation, particularly at positions C1235, C1236, C1240, and C1241, has the most significant impact on viral particle infectivity. This modification acts by influencing the formation of the S1/S2 subunits of the S protein [21] (Figure 2A). C1247 and C1248, although also among the six high-level palmitoylation sites, are located in a more distal region of the cytoplasmic tail. Mutation of these two sites has a comparatively minor effect on pseudovirus infectivity, indicating that efficient palmitoylation does not always translate to a dominant functional role in viral entry [22].

### 2.2. Replication

During SARS-CoV-2 assembly, the envelope protein (E protein) and membrane protein (M protein) are two key structural proteins that jointly regulate the maturation and localisation of the S protein through different mechanisms, consequently facilitating the efficient assembly of SARS-CoV-2 particles [33]. The M protein and E protein work together—the former through direct interaction and the latter through indirect regulation of the secretory pathway—to ensure that the S protein stays at the correct location, consequently facilitating efficient SARS-CoV-2 assembly [34]. The M protein interacts directly with the S protein. Recognition of this interaction depends on a specific dibasic retrieval signal at the C-terminus of the S protein’s cytoplasmic tail, whose core sequence is KxHxx. Here, “K” stands for the basic amino acid lysine (Lys), ‘H’ stands for the basic amino acid histidine (His), and “x” represents any amino acid [35]. The paired occurrence of charged amino acids (Lys and His) is a key feature of this signal, enabling it to be recognised and bound by the Coatomer Protein Complex I (COPI) on the endoplasmic reticulum membrane within the host cell’s endomembrane transport system [36]. This motif helps the S protein become retained in the endoplasmic reticulum–Golgi intermediate compartment (ERGIC), thereby increasing the chance of encountering the M protein, which is also localised there [37]. The E protein, acting as a SARS-CoV-2 pore protein, indirectly causes the S protein (and other glycoproteins) to be retained within the cell by slowing down the overall rate of the cellular secretory pathway. This process does not depend on the S protein’s own recycling signal [38].

These M/E-mediated mechanisms govern where the S protein is retained and how it accumulates at assembly sites. Palmitoylation acts at an earlier, complementary step: by protecting newly synthesized S from ER-associated degradation and promoting ordered lipid-domain formation in the ERGIC, it increases the amount of functional S protein available for M/E-mediated assembly. Thus, M/E interactions and S palmitoylation are distinct but convergent processes that together support efficient SARS-CoV-2 particle assembly. Palmitoylation is critical for the stability of newly synthesised S protein in the endoplasmic reticulum (ER). S proteins lacking palmitoylation are more readily recognised and degraded by the endoplasmic reticulum-associated protein quality control system, consequently reducing the total amount of S proteins that can fold correctly and enter the subsequent secretory pathway [39]. This suggests that palmitoylation plays a role in protecting the S protein and promoting its effective biosynthesis [32].

The cytoplasmic tail region contributes to the stabilisation of the three-dimensional structure of the S-protein homotrimer through palmitoylation [40]. Trimers are key viral virulence factors, and their stability is critical for membrane fusion and SARS-CoV-2 infectivity. A deficiency in palmitoylation reduces the formation of S-protein trimers, thereby decreasing the efficiency of viral particle assembly and infectivity [41]. In addition, palmitoylation may facilitate the formation of cholesterol-enriched lipid domains within the viral envelope, thereby promoting virus assembly [42].

In the SARS-CoV-2 assembly pathway, the upper pathway is designed to produce and release intact progeny SARS-CoV-2 particles: after the newly synthesised S protein is generated in the endoplasmic reticulum, it is transported to the Golgi apparatus by COPII-coated vesicles derived from the endoplasmic reticulum. Subsequently, the critical COPI vesicles transport them retrograde from the Golgi apparatus to the endoplasmic reticulum–Golgi intermediate compartment, which serves as the factory for SARS-CoV-2 assembly [43]. Here, the S protein combines with components such as the viral genome to form new viruses, which are ultimately released via exocytosis. In cell surface expression, the lower pathway, along with the direct transmission pathway, represents a more efficient “shortcut”: during transport, portions of the S protein are recycled from endosomes by the SNX27 protein and transported to the cell membrane surface. These S proteins on the cell surface can bind to ACE2 receptors on adjacent healthy cells, directly mediating cell membrane fusion and forming multinucleated syncytia. This direct transmission between cells allows the virus to spread without forming complete SARS-CoV-2 particles. This palmitoylation modification plays a critical role in the entire process. Host DHHC palmitoyltransferases attach palmitic acid to cytosol-facing cysteines of the S protein via a thioester bond [22]; this covalent lipid addition increases the local hydrophobicity of the cytoplasmic tail. In SARS-CoV-2, ZDHHC20 and ZDHHC9 sequentially modify ten C-terminal cysteines of the S protein, and N-terminal C15 palmitoylation has also been reported; this modification reduces ER-associated degradation of nascent S protein, thereby stabilising newly synthesised S and promoting its correct trimerisation [31,44]. By inserting long-chain fatty acids into the inner leaflet, palmitoylated S protein further drives formation of cholesterol- and sphingolipid-rich ordered nanodomains in the ERGIC, ensuring its proper anchoring in cholesterol-rich membrane environments where viral assembly and membrane fusion occur [44]. This is important for both the efficient assembly of SARS-CoV-2 in the ERGIC and the S protein-mediated membrane fusion process [32] (Figure 2B).

### 2.3. Release

The S protein of COVID-19 is palmitoylated at its cytoplasmic tail, a process catalysed by host-cell proteins such as DHHC9 [45]. This modification is not essential for the transport of the S protein to the cell membrane or its binding to the ACE2 receptor, but it plays the following critical roles. First, the palmitoyl group acts as a “fatty anchor,” firmly anchoring the tail of the S protein to the viral envelope, thereby providing mechanical support for the conformational changes that occur during membrane fusion; second, palmitoylation promotes the aggregation of S proteins in lipid rafts on the cell membrane, increasing their local density at the fusion site. Together, these effects optimise the fusion activity of the S protein, enabling it to efficiently mediate membrane fusion between the virus and the host cell [46]. If palmitoylation is inhibited, although SARS-CoV-2 particles can still be assembled and released, the S proteins on their surfaces become unstable, with anchoring and dispersed distribution, severely impairing their ability to drive membrane fusion, which markedly reduces the infectivity of the offspring SARS-CoV-2 [47].

Palmitoylation of the S protein has been shown to promote S-mediated syncytia formation and cell–cell fusion, processes that underlie direct cell-to-cell spread of SARS-CoV-2 from infected to uninfected cells [31,48]. The S protein binds to ACE2 receptors on adjacent uninfected cells, promoting fusion of cell membranes to form multinucleated syncytia [49]. This allows the virus to spread between cells without releasing intact particles, resulting in more widespread tissue damage [32].

Experiments have shown that palmitoylation-deficient S protein mutants mediate significantly reduced syncytium formation, and all mutants with all cysteines in the cytoplasmic tail region replaced are completely unable to form syncytia [42]. This suggests that palmitoylation is a key mechanism by which the virus achieves direct cell-to-cell transmission by regulating the distribution and fusion activity of the S protein on the plasma membrane.

On the other hand, the function of the SARS-CoV-2 envelope protein (E protein) also depends on palmitoylation [50]. The envelope protein forms a pentameric ion channel on the viral envelope; its main role is to act as a proton (H^+^) channel, transporting H^+^ from the lumen of organelles such as the Golgi apparatus into the cytoplasm, thereby raising the lumen pH. This alkaline environment helps protect the S protein and promotes the virus’s release from infected cells, which is key to its successful exit. Studies have shown that palmitoylation is important for the proper localisation and stability of the E protein on the cell membrane. A lack of palmitoylation impairs the membrane localisation of the envelope protein. This causes the ion channels it forms to become unstable, with reduced pore size or even collapse, consequently disrupting their proton-conducting function and thereby inhibiting viral release [51].

To summarise, the SARS-CoV-2 “hijacks” the host’s palmitoylation system to optimise both the membrane fusion activity of the S protein and its ability to spread between cells, while guaranteeing the functional integrity of the envelope protein’s ion channel. This synergistically increases the virus’s infectivity and release efficiency, representing a key strategy by which SARS-CoV-2 exploits host mechanisms to facilitate its own replication and transmission (Figure 2C).

## 3. Hosts Use Palmitoylation as a Defence Mechanism

### 3.1. Activation of Innate Immune Signalling Pathways

#### 3.1.1. MAVS-Mediated Antiviral Signaling Pathways

**Figure 3:**
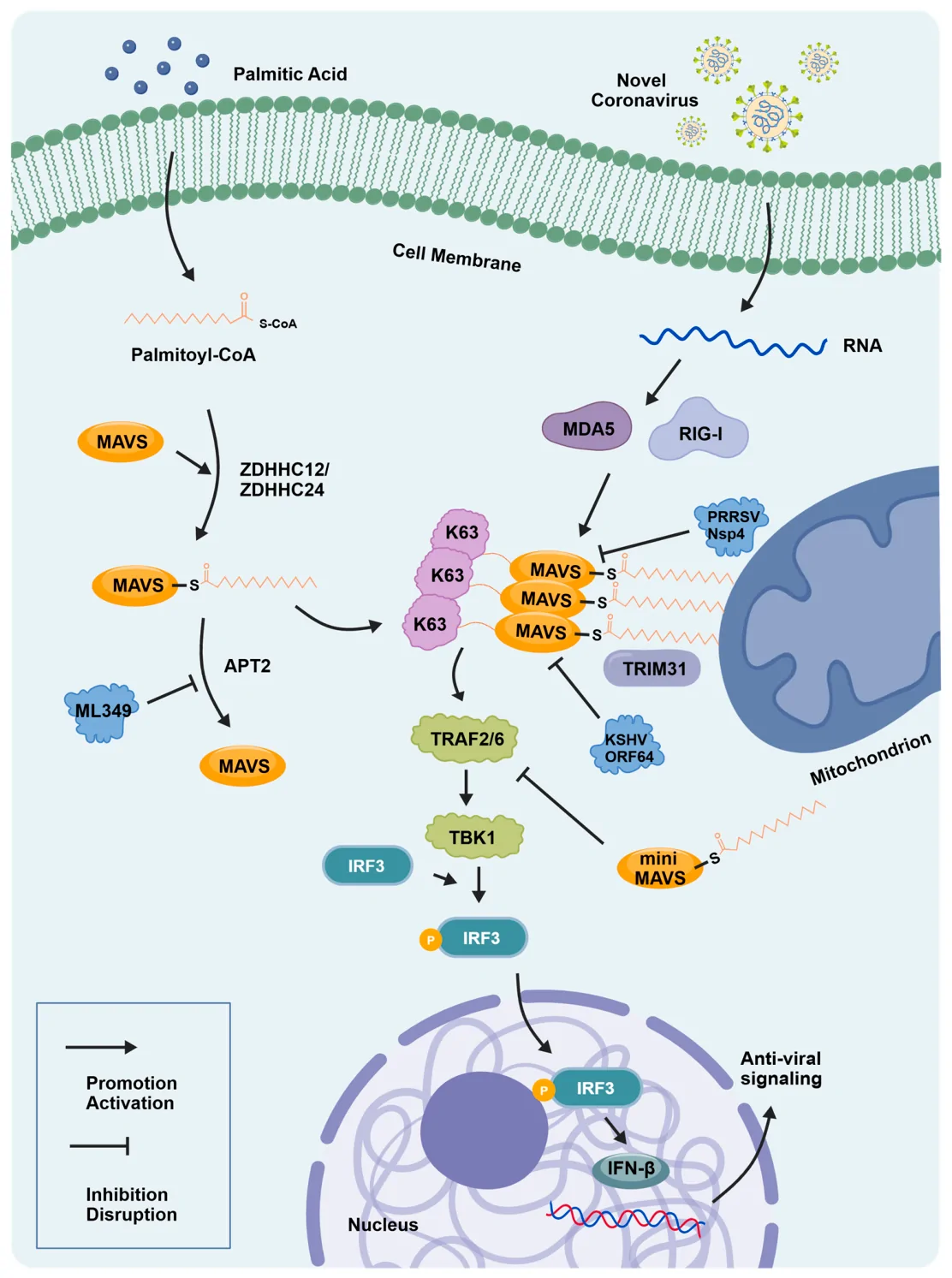
MAVS-mediated palmitoylation in antiviral innate immunity and its regulation. Palmitic acid is converted to palmitoyl-CoA in the cytoplasm. ZDHHC12/24 catalyses MAVS palmitoylation, promoting its prion-like aggregation on mitochondria—the functional core for signalling. Upon detection of viral RNA by RIG-I/MDA5, palmitoylated MAVS aggregates recruit TBK1 and assemble signalling complexes. This activates the TBK1-IRF3-IFN axis: TBK1 phosphorylates IRF3, which translocates to the nucleus to induce type I interferons (e.g., IFN-β). Meanwhile, TRIM31-mediated K63 ubiquitination of MAVS synergises with palmitoylation, recruiting TRAF2/6 to amplify TBK1 activation. Negative regulation: viral proteins (e.g., PRRSV Nsp4, KSHV ORF64) directly inhibit MAVS; Mini-MAVS competes for recruitment of TRAF2/6. The APT2 agonist ML349 promotes MAVS depalmitoylation, disassembles aggregates, and terminates signalling. Note: RIG-I, retinoic acid-inducible gene I; MDA5, melanoma differentiation-associated protein 5.

Palmitoylation is a core regulatory mechanism controlling the antiviral signalling function of Mitochondrial Antiviral Signalling Protein (MAVS) and a critical step for activating antiviral defence [52]. See the figure for the detailed mechanism (Figure 3). This modification, catalysed by the palmitoyl transferase ZDHHC24, anchors MAVS to the mitochondrial membrane and induces the formation of prion-like functional aggregates [53]. This is a necessary condition for activating the downstream TRAF family member-associated NF-kappa-B activator (TANK)-binding kinase 1–interferon regulator factor 3 (TBK1–IRF3) signalling pathway and inducing the production of type I interferons [54]. The palmitoylation-deficient mutant (C79S) or the polymerisation-deficient mutant (W56R) fails to form effective aggregates and cannot efficiently activate Type I Interferon Beta (IFN-β) [55].

This modification process underpins MAVS’s efficient signalling transduction. Modified MAVS binds more effectively to upstream receptors (RIG-I/MDA5) and the downstream kinase TBK1, consequently promoting the formation of functional signal transduction complexes [56]. This effectively activates the TANK-binding kinase 1–interferon regulatory factor 3–interferon (TBK1-IRF3-IFN) signalling pathway, thereby enhancing the host’s innate antiviral immune response [14]. Studies have shown that palmitoylation of MAVS is in a cascade relationship with another post-translational modification—K63-linked ubiquitination [57]. Specifically, TRIM31-mediated K63 ubiquitination serves as an upstream signal for palmitoylation [58]. Furthermore, the palmitoylation level of the MAVS ubiquitination site mutant (K0) is significantly reduced, and this reduction cannot be rescued by ZDHHC12 overexpression. It can recruit ZDHHC12 and bind to MAVS, thereby catalysing MAVS palmitoylation and forming a “ubiquitination–palmitoylation” regulatory cascade [55].

Within cells, the palmitoylation level of MAVS is dynamically regulated by a specific enzymatic system to control the antiviral immune response precisely [59]. The palmitoyl transferase ZDHHC24 is responsible for catalysing this modification to activate signalling, while the depalmitoylase APT2 negatively regulates the process by removing this modification, thereby preventing excessive immune responses [60]. Therefore, either inhibiting APT2 (e.g., using ML349) or increasing ZDHHC24 activity through dietary supplementation with palmitic acid or a high-fat diet can effectively increase MAVS palmitoylation levels, thereby considerably enhancing the host’s overall antiviral immunity [53].

#### 3.1.2. The cGAS-STING Pathway

**Figure 4:**
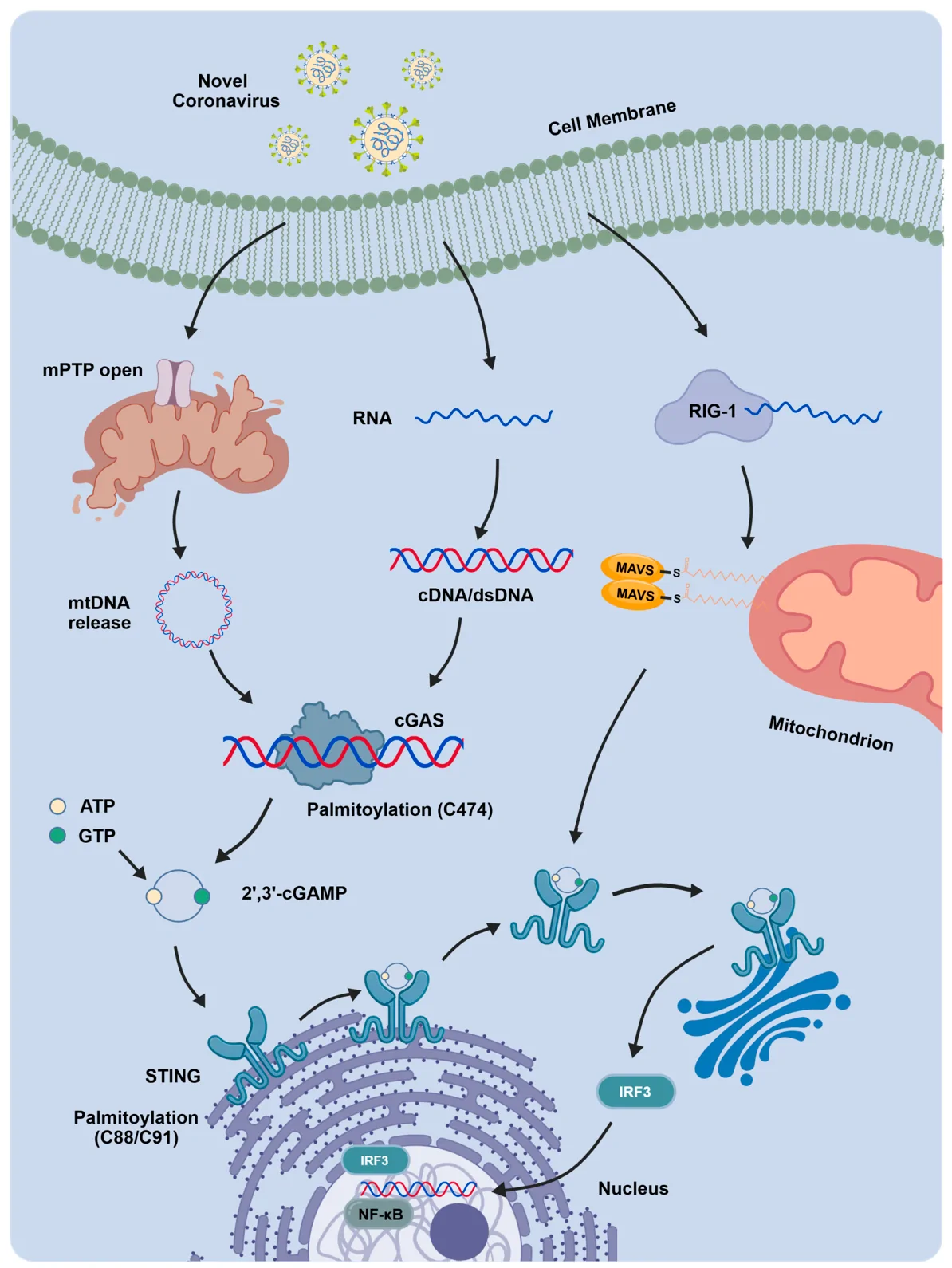
Host cells recognise SARS-CoV-2 nucleic acids via the cGAS-STING pathway to induce antiviral immunity. Two main mechanisms are shown: Pathway 1: cGAS recognises cytoplasmic DNA. SARS-CoV-2 infection triggers mitochondrial damage and the release of mtDNA into the cytoplasm. Viral RNA may also generate complementary or double-stranded DNA via reverse transcriptase activity. Cytoplasmic DNA is bound by cGAS, which then synthesises 2′3′-cGAMP from GTP and ATP. Pathway 2: STING activation and downstream signalling. cGAMP binds ER-localized STING, inducing its conformational change and oligomerisation. Activated STING translocates through the Golgi apparatus, recruiting and activating TBK1. TBK1 phosphorylates IRF3, which dimerises and enters the nucleus to bind interferon-stimulated response elements, triggering type I interferon production. cGAS and STING are annotated with their respective palmitoylation sites (C474 for cGAS; C88/C91 for STING).

The stimulator of interferon genes (STING, also known as MITA, TMEM173, MPYS, and ERIS) is an endoplasmic reticulum-anchored protein whose structure comprises four transmembrane segments and a cytoplasmic domain responsible for ligand binding and signal transduction [61,62]. See the figure for the detailed mechanism (Figure 4). This cytoplasmic domain typically exists as a dimer, and its dimerisation is critical for activation. Upon binding to the second messenger cyclic GMP-AMP (cGAMP), the cytoplasmic domain of STING experiences a substantial conformational change, thereby activating downstream signalling [63,64]. Palmitoylation of human STING at Cys88 and Cys91 is a key modification for its activation and signal transduction efficiency [65]. Among these, Cys88 is located in the linker region between STING’s second and third transmembrane segments (TM2-TM3) and is buried within the protein, whereas Cys91, being exposed on the surface, serves as the primary palmitoylation site; its modification may enhance STING activation by influencing the formation and robustness of the tetramer interface [62]. This palmitoylation modification occurs after STING is transported to the Golgi apparatus and acts as a key regulatory step in its transport from the endoplasmic reticulum to the Golgi apparatus and its functional localisation [66,67]. The use of palmitoylation inhibitors (such as 2-bromopalmitate) effectively inhibits this modification, thereby eliminating the downstream type I interferon response [62].

The cGAS-STING pathway is triggered by abnormal cytoplasmic DNA (such as mitochondrial DNA or micronuclei) and can induce both type I interferon and autophagy, two antiviral responses [68]. Among these, autophagy is regarded as an evolutionarily conserved, interferon-independent primitive function that acts as a key mechanism for suppressing SARS-CoV-2 replication [69]. This pathway possesses multiple layers of negative regulatory mechanisms: on the one hand, the inhibition of cGAS function—specifically, the palmitoylation of cGAS at position C474 (catalyzed by ZDHHC18)—reduces its affinity for DNA, impedes oligomerization, and thereby suppresses its enzymatic activity and downstream signaling, constituting a form of negative regulation [70]. On the other hand, to inhibit STING activation, small-molecule compounds such as 4-octyl itaconate (4-OI) can alkylate the Cys91 site of STING, thereby inhibiting its palmitoylation and subsequent oligomerization, and thus negatively regulating the activation of the entire pathway [62]. Together, these findings demonstrate that the cGAS-STING pathway is directly regulated by S-palmitoylation at multiple levels: cGAS activity is suppressed by its own palmitoylation, while STING activation requires palmitoylation at the Golgi. This dual regulation establishes a clear link between palmitoylation and cGAS-STING signaling.

The ORF3a protein of SARS-CoV-2 is a key immune evasion protein that specifically inhibits the antiviral autophagy function induced by the cGAS-STING pathway, while not affecting the pathway’s ability to induce type I interferons [71]. This mechanism includes ORF3a binding to the STING protein, thereby blocking the interaction between STING and the key autophagy protein microtubule-associated protein 1 light chain 3 (LC3), inhibiting the antiviral autophagy flux, and consequently promoting SARS-CoV-2 replication. This function is a capability that SARS-CoV-2 acquired through its development from bat coronaviruses [72]; its inhibitory potency is significantly greater than that of the open reading frame 3a (ORF3a) protein of SARS-CoV, which may have enhanced the virus’s transmissibility. This finding also suggests that ORF3a is a potential target for antiviral drugs [69].

### 3.2. Augmenting the Functionality of Restriction Factors

The antiviral activity of IFITM3 depends on the palmitoylation of a cysteine residue on its adjacent amphipathic helix; this is currently the only post-translational modification known to increase its antiviral activity [73]. In terms of host defence, the S-palmitoylation of IFITM3 is key to its potent antiviral activity. This modification occurs at three cysteine residues in IFITM3 and positively regulates its antiviral function [74].

Research has found that the palmitoyltransferase ZDHHC20 plays a key role in this process [75]. In overexpression experiments, ZDHHC20 significantly increased IFITM3 palmitoylation and was the only factor capable of enhancing its antiviral activity; this effect was strictly dependent on its enzymatic activity. Its catalytic-site mutant, ZDHHC20-C156A, neither promotes palmitoylation nor enhances antiviral restriction [73]. However, multiple ZDHHC enzymes can catalyse the palmitoylation of IFITM3, and this excess ensures the robustness of the antiviral response [76]. In ZDHHC20-knockout cells, knockdown of other ZDHHC enzymes still reduces the palmitoylation of endogenous IFITM3, suggesting that under physiological conditions, multiple ZDHHC enzymes, including ZDHHC20, collectively maintain the activated state of IFITM3 [73].

Specifically, S-palmitoylation enhances IFITM3’s ability to inhibit SARS-CoV-2 fusion with endosomal membranes by regulating its subcellular localisation, stability, interaction with cholesterol, and molecular conformation, thereby preventing SARS-CoV-2 from releasing its genetic material into the cytoplasm. The article notes that several enzymes in the ZDHHC family are involved in the palmitoylation of IFITM3 [74].

## 4. Prospects for Focused Therapeutic Strategies for Palmitoylation

### 4.1. Targeting SARS-CoV-2 Proteins

Targeting the palmitoylation of SARS-CoV-2 proteins constitutes a promising antiviral strategy. Research has shown that palmitoylation of host enzymes plays a key role in the life cycle of various coronaviruses [77]. Palmitoylation of the SARS-CoV-2 S protein is believed to facilitate its merging with the host cell membrane, clearly indicating that ZDHHC5 is a possible therapeutic target for inhibiting SARS-CoV-2 and other coronaviruses [78]. Similarly, in porcine epidemic diarrhoea virus (PEDV), ZDHHC5-mediated palmitoylation of the S protein improves its stability by preventing recognition by heat shock cognate protein of 70 kDa (HSC70) and resisting degradation via molecular chaperone-mediated autophagy (CMA), thereby promoting viral transmission. These research findings highlight the important role of palmitoylation in coronavirus propagation and support the use of palmitoylation as a promising target for developing antiviral approaches [79].

Interventions targeting S protein palmitoylation can effectively inhibit SARS-CoV-2 infection [80]. Experiments have shown that knockdown of ZDHHC5 expression using short hairpin RNA (shRNA) reduces palmitoylation of the SARS-CoV-2 S protein and leads to significant defects in S protein-mediated cell–cell fusion and pseudovirus entry. Consistent with this, treatment with the broad-spectrum protein palmitoylation inhibitor 2-bromopalmitate (2-BP) also reduced palmitoylation levels of the S protein and inhibited S protein-mediated membrane fusion and the infectivity of SARS-CoV-2 pseudoviruses; furthermore, no cytotoxicity was observed under the experimental conditions. These results suggest that interfering with the palmitoylation of the S protein helps limit the transmission of SARS-CoV-2 [48].

In addition, palmitoylation of other coronavirus proteins also exhibits antiviral effects. Reflecting a conserved dependency in coronaviruses, Middle East respiratory syndrome coronavirus (MERS-CoV) inhibiting the activity of acyl-CoA carboxylase in host cells or directly inhibiting protein palmitoylation leads to impaired viral particle release and the accumulation of nucleocapsids during the early stages of viral assembly, indicating that the viral assembly process depends on lipid metabolism and specific protein modifications. Studies have confirmed that palmitoylation of the MERS-CoV S and E proteins plays a critical role in viral particle assembly and maturation. That exogenous palmitic acid supplementation can reverse these inhibitory effects provides further experimental evidence for antiviral strategies targeting this post-translational modification [81].

### 4.2. Targeting Host Receptors

Research targeting host receptors is an important strategy in antiviral drug development. Among these studies, research centred on angiotensin-converting enzyme 2 (ACE2) as a key host receptor for the novel coronavirus has made significant progress. In one study, researchers engineered a fusion protein—PM-ACE2—by fusing ACE2 with an S-palmitoyl-dependent plasma membrane targeting sequence (amino acids 1–11 of GAP43, MLCCMRRTKQV), causing it to enrich on the surface of extracellular vesicles (EVs), thereby generating extracellular vesicles modified with the plasma membrane-targeted ACE2 fusion protein (PM-ACE2-EVs) [82]. The palmitoylation of the cysteine residues within this GAP43-derived motif serves as a fatty acid membrane anchor that drives PM-ACE2 to the plasma membrane and promotes its efficient packaging into EVs, resulting in a ~7-fold enrichment of ACE2 on the EV surface compared to ACE2-WT. This high-density display of ACE2 enables PM-ACE2-EVs to bind the SARS-CoV-2 spike RBD with high affinity and competitively block spike engagement with cellular ACE2, achieving significantly stronger neutralization potency than unmodified ACE2-Evs [82]. In vitro, these vesicles bind with high affinity to the receptor-binding domain (RBD) of the SARS-CoV-2 S protein, effectively blocking the virus’s interaction with ACE2 on the cell surface. In experiments with human ACE2-transgenic mice, PM-ACE2-Evs demonstrated neutralising activity against both pseudoviruses and SARS-CoV-2, effectively suppressing viral load and thereby protecting the host from SARS-CoV-2-induced pulmonary inflammation. Further mechanistic investigations revealed that pretreatment of Vero E6 clone cells derived from African green monkeys with the broad-spectrum palmitoylation inhibitor 2-bromopalmitate (2-BP) before treatment with these cells significantly inhibited the production of ACE2-positive EVs. In contrast, cells pretreated with ML348, a selective inhibitor of the de-palmitoylating enzyme acyl-protein thioesterase 1 (APT1), showed significantly increased secretion of ACE2-containing extracellular vesicles. This reveals the critical role of protein palmitoylation in regulating the transport of ACE2 into EVs. Based on this finding, ACE2-rich EVs developed as a “decoy” therapy can inhibit infection by competitively binding to the SARS-CoV-2 [82].

In addition to directly utilising or modifying host receptors as “decoys,” targeting the regulatory enzymes that modify host receptors or their associated factors is another important approach. A proof of concept for this strategy is provided by research on the porcine acute diarrhoea syndrome coronavirus (SADS-CoV), which has revealed that the host zinc finger DHHC-type palmitoyl transferase 17 (ZDHHC17/ZD17) is a key factor in its infection. Functional studies indicate that ZD17 primarily supports viral genome replication through its palmitoylation activity, without affecting other processes such as SARS-CoV-2 entry. The broad-spectrum palmitoylation inhibitor 2-BP effectively inhibited SARS-CoV-2 replication in a dose-dependent manner across various cell types, confirming that interfering with palmitoylation can suppress SARS-CoV-2 infection. The study indicates that ZD17 is a potential host-directed therapeutic target. Although 2-BP exhibits antiviral activity, its broad-spectrum nature may lead to side effects; therefore, the development of small-molecule inhibitors that specifically target ZD17 represents a promising direction for future therapies against SARS-CoV-2 [83].

In summary, these studies demonstrate therapeutic strategies targeting host receptors from various perspectives: first, by engineering host receptors (such as ACE2) into “decoys” (such as EVs) with similar membrane topologies to neutralize SARS-CoV-2 directly; second, by inhibiting or regulating the activity of host enzymes critical for host receptor function or SARS-CoV-2 replication (such as palmitoyl transferase ZD17), the SARS-CoV-2 infection cycle is indirectly disrupted. Both strategies bypass SARS-CoV-2 itself, which mutates frequently, and instead target relatively conserved host proteins, offering a new approach to addressing SARS-CoV-2 resistance [84].

### 4.3. Modulation of Host Immunity

Modulation of host immunity can be achieved through the synergistic integration of two major approaches: biological metabolic regulation and precise chemical structural design. At the metabolic level, physical activity can activate the AMP-activated protein kinase (AMPK) pathway, thereby inhibiting acetyl-CoA carboxylase and directly reducing palmitate synthesis [85,86]. Given that the palmitoylation of the SARS-CoV-2 S protein is critical for membrane fusion and the infectivity of viral particles, this mechanism reduces the substrates required for viral replication [87]. At the same time, exercise can enhance the bioavailability of nitric oxide, thereby providing a specific defence against viruses through synergistic effects [88]. Pharmacological interventions, such as orlistat, can also interfere with fatty acid synthesis by inhibiting fatty acid synthase, thereby offering an alternative approach to metabolic regulation [89].

Beyond reducing the supply of palmitate, another strategy is to optimize the molecular structures that utilize palmitoyl groups for immune stimulation. Precise control of stereochemistry is a key strategy for designing highly effective immune adjuvants. Recent studies have shown that chiral, all-R-configuration N-tetradecyl carbamoyl peptide (Upam) derivatives offer significant advantages in activating immune responses [90]. This advantage is substantiated by data showing that, in inducing dendritic cell maturation and the production of the p40 subunit of interleukin-12 (IL-12p40), the activity of R-UPam-Ser was comparable to that of the diastereomeric mixture but was significantly superior to its S-epimer counterpart. In terms of specific activation of Toll-like receptor 2 (TLR2), R-configuration ligands exhibit an even stronger activating capacity than the mixture [91]. Structural optimisation further revealed that derivatives such as R-UPam-Dab and R-UPam-Abu exhibit immunostimulatory activity comparable to or superior to that of the parent compounds [92]. Notably, many of these optimized immunostimulatory molecules contain palmitoyl moieties, whose spatial configuration critically influences their receptor-binding affinity.

This principle is exemplified by the natural immune adjuvant S-[2,3-bis(palmitoyloxy)propyl]-L-cysteine (Pam3Cys) which has a glycerol moiety that is inherently in the R-configuration; however, racemic mixtures have often been used in the past for the sake of synthetic convenience [93]. Previous studies have demonstrated that the pure R-enantiomer is superior to the S-enantiomer and mixtures thereof in both inducing cytokine responses and promoting antibody production. From a molecular mechanism perspective, its advantage may stem from the optimised hydrogen-bond network formed between specific groups in the R-conformation and key residues (such as Phe-312) within the binding pocket of Toll-like receptor 1 (TLR1), thereby enabling more stable and efficient specific binding [92].

Taken together, these findings highlight two complementary strategies centered on palmitoylation-related immune modulation. On one hand, modulating host metabolic pathways through exercise or pharmacological interventions can reduce the supply of palmitate required for viral spike protein palmitoylation, thereby limiting viral infectivity. On the other hand, at the level of chemical design, optimizing the stereochemistry of palmitoyl-containing immune adjuvants enables more precise and efficient activation of TLR-mediated immune responses. Both strategies—targeting the host’s internal metabolic environment and engineering exogenous immune stimulants—converge on the central role of palmitoylation in regulating antiviral immunity, and together provide important directions for controlled immune regulation. Table 1 summarises the current classifications of COVID-19 targeted treatment strategies and their corresponding mechanisms of action.

## 5. Summary and Outlook

Palmitoylation serves as a critical link between protein function and the cellular membrane lipid environment, playing a dynamic, bidirectional role in host–pathogen interactions. This article systematically elucidates the dual role of palmitoylation in SARS-CoV-2 infection [96].

On the one hand, SARS-CoV-2 “hijacks” the host cell’s palmitoylation system to modify its own key proteins, thereby promoting the infection cycle. The palmitoylation of the S protein is fundamental to SARS-CoV-2’s efficient entry, stable assembly within the cell, and effective release, while the palmitoylation of the envelope protein is critical to its ion channel function and SARS-CoV-2 release. On the other hand, the host uses the same system to tune its antiviral defences finely. The palmitoylation of key immune adapter molecules, such as MAVS (which requires palmitoylation for its aggregation and downstream signaling) and STING (requiring S-palmitoylation at Cys88/Cys91 for Golgi activation), serves as a central switch for their activation, localisation, and signal transduction, driving the production of type I interferons and antiviral autophagy. At the same time, the antiviral activity of interferon-induced transmembrane protein 3 is strictly dependent on its palmitoylation [97]. This “attack–defence contest” over the same biochemical process provides profound insight into the coevolutionary struggle between host and pathogen at the molecular level.

Based on the aforementioned mechanisms, targeting palmitoylation shows multifaceted potential for antiviral therapy [98]. Strategies include: developing specific inhibitors that target host palmitoyl transferases (such as ZDHHC5/9/20) modified by SARS-CoV-2 proteins; reducing the substrates required for SARS-CoV-2 modification through metabolic interventions (such as modulating fatty acid synthesis); and competitively blocking SARS-CoV-2 infection through engineering approaches (such as preparing palmitoylation-dependent ACE2 “decoy” vesicles).

Future research should focus on addressing the following key issues:

First, site-specific functional map: It is currently known that the S protein possesses as many as 10 palmitoylation sites, far exceeding the number found in typical membrane proteins. Using techniques such as CRISPR screening and site-specific mutagenesis, we aim to precisely elucidate the functions of each palmitoylation site on SARS-CoV-2 and key host proteins [99].

Second, dynamic competition and resource acquisition: SARS-CoV-2 and hosts engage in intense competition for limited palmitic acid and enzyme resources. Research should focus on how SARS-CoV-2 hijacks modification resources by regulating the transcription or activity of host DHHC enzymes (such as ZDHHC20), thereby suppressing the function of host antiviral proteins (such as IFITM3), and elucidating the molecular mechanisms underlying this “battle for resources”.

Third, moving toward Precision Therapy: Given the high toxicity of broad-spectrum inhibitors (such as 2-BP), future treatment strategies should shift in this direction. This includes developing highly selective inhibitors targeting specific DHHC enzymes (such as ZDHHC20) or utilising engineered exosome delivery technology to inhibit SARS-CoV-2 replication while avoiding interference with the host’s normal immune signalling pathways, thereby achieving a true “precision strike”.

## Figures and Tables

**Table 1:** Classification of Strategies for Targeted Treatment of COVID-19.

Targeted Strategy	Medications/Interventions	Target/Mechanism	References
targeting viral proteins	2-Bromopalmitate (2-BP)	Broad-spectrum inhibition of the ZDHHC family, blocking S-protein palmitoylation, and disrupting membrane fusion	[47]
	ZDHHC20 inhibitor (screening compound)	Specifically inhibits ZDHHC20, the primary modifier of S protein, thereby reducing viral replication	[44]
	Cinnamic acid derivatives (such as CAPE)	Inhibiting ZDHHC20 activity disrupts S-protein palmitoylation and cholesterol binding	[47]
	PLA2 inhibitors (such as AACOCF3)	Inhibiting phospholipase A2, altering the membrane lipid environment, and indirectly influencing S-protein modification	[94]
targeting host receptors	ML348	Inhibition of ZDHHC3 reduces ACE2 palmitoylation and membrane localization	[82]
	2-BP	Inhibits ACE2 palmitoylation and reduces its accumulation in exosomes	[82]
	Engineering PM-ACE2-EVs	Neutralizing Viruses by Using Palmitoylated ACE2 as a “Virus Trap”	[82]
modulating the host immune response	ML349	Inhibiting the de-palmitoylase APT2 to enhance the MAVS signaling pathway	[53]
	Disulfiram	Inhibiting NLRP3 palmitoylation (Cys126) to mitigate the inflammatory storm	[95]
	Palmitic acid (PA)	Promotes MAVS palmitoylation (mediated by ZDHHC24), activating IFN	[53]
	ZDHHC4/7/12 activator	Promotes MAVS aggregation and enhances innate antiviral immunity	[55]

## Data Availability

No new data were created or analyzed in this study. Data sharing is not applicable to this article.

## Abbreviations

SARS-CoV-2	severe acute respiratory syndrome coronavirus 2
ZDHHC	zinc finger-containing DHHC
ZDHHCs	a family of zinc finger DHHC domain-containing protein palmitoyl transferases
MAVS	mitochondrial antiviral signaling protein
STING	stimulator of interferon genes
IFITM3	interferon-induced transmembrane protein 3
ACE2	angiotensin converting enzyme 2
PTM	post-translational modification
PATs	palmitoyl transferases
APTs	acyl-protein thioesterases
PPTs	palmitoyl protein thioesterases
S Protein	spike protein
PA	palmitic acid
DNL	de novo lipogenesis
COVID-19	coronavirus disease 2019
RBD	receptor binding domain
HR	heptad repeats 1 and 2
M2 Protein	M2 ion channel protein
HA Protein	hemagglutinin protein
E Protein	envelope protein
M Protein	membrane protein
ERGIC	endoplasmic reticulum–Golgi intermediate compartment
ER	endoplasmic reticulum
COPI	Coatomer Protein Complex I
COPII	Coat Protein Complex II
IFN-β	Type I Interferon Beta
TBK1-IRF3	TANK-binding kinase 1–Interferon regulatory factor 3
TBK1-IRF3-IFN	TANK-binding kinase 1–Interferon regulatory factor 3–Type I Interferon
cGAMP	cyclic GMP-AMP
TM2-TM3	t he connecting region from the second transmembrane segment to the third transmembrane segment
4-OI	4-Octyl Itaconic Acid
LC3	light chain 3
ORF3a	open reading frame 3a
PEDV	porcine epidemic diarrhea virus
HSC70	heat shock cognate protein of 70 kDa
CMA	chaperone-mediated autophagy
shRNA	short hairpin RNA
2-BP	2-bromopalmitate
MERS-CoV	Middle East respiratory syndrome coronavirus
EVs	Extracellular vesicles
PM-ACE2-EVs	Plasma membrane-targeted ACE2 fusion protein-engineered extracellular vesicles
APT1	acyl protein thioesterase 1
SADS-CoV	porcine acute diarrhea syndrome coronavirus
ZD17	zinc finger DHHC-type palmitoyltransferase 17
AMPK	AMP-activated protein kinase
Upam	N-tetradecylaminocarbonyl lipopeptide
IL-12p40	the p40 subunit of interleukin-12
TLR1/2	toll-like receptor 1/2
Pam3Cys	S-[2,3-di(palmitoyloxy)propyl]-cysteine
RIG-I	Retinoic acid-inducible gene I
MDA5	Melanoma differentiation-associated protein 5
TANK	TRAF family member-associated NF-kappa-B activator
